# P-810. Blood Culture Stewardship Efforts at a Comprehensive Cancer Center Reduced Isolation of Skin Flora Contaminants Without Compromising Patient Care

**DOI:** 10.1093/ofid/ofaf695.1018

**Published:** 2026-01-11

**Authors:** Jovan Borjan, Micah M Bhatti, Guy Handley, Nancy N Vuong, Amy Spallone

**Affiliations:** The University of Texas MD Anderson Cancer Center, Houston, TX; The University of Texas MD Anderson Cancer Center, Houston, TX; The University of Texas MD Anderson Cancer Center, Houston, TX; The University of Texas MD Anderson Cancer Center, Houston, TX; University of Texas MD Anderson Cancer Center, Houston, Texas

## Abstract

**Background:**

A global supply chain shortage led hospitals to establish conservation plans for Becton Dickson (BD) BACTEC blood culture bottles. Results and safety outcomes of these conservation efforts in immunocompromised patients are lacking. The objective of this study was to describe the outcomes of blood culture (BCx) stewardship interventions aimed at conserving BCxs during the shortage while preserving patient care and safety.

**Figure:**
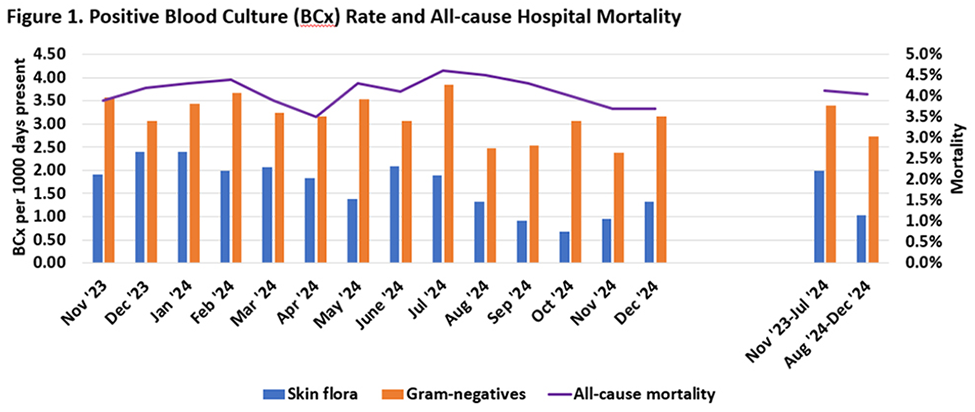

**Figure:**
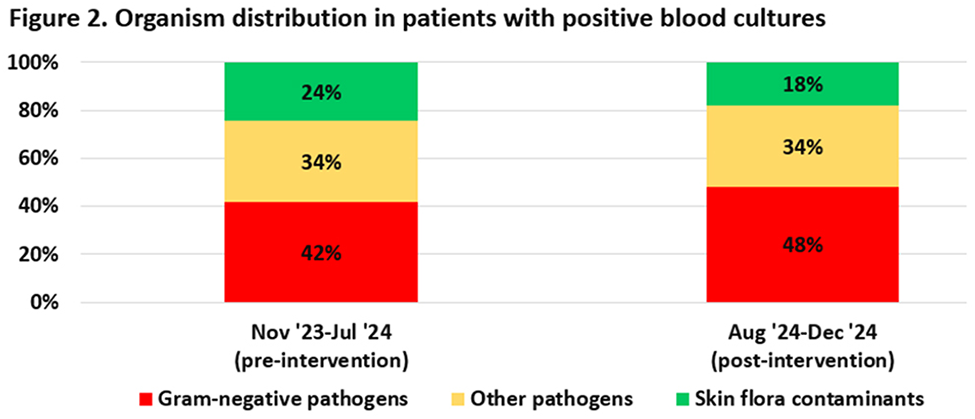

**Methods:**

This is a retrospective, single-center study evaluating BCx utilization, positivity rates, organism distribution, and corresponding all-cause hospital and acute cancer care center (ACCC) mortality pre-intervention (11/1/2023 - 7/31/2024) and post-intervention (8/1/2024 - 12/31/2024). Diagnostic stewardship interventions included changes to blood culture ordering in the electronic health record (EHR), changes to ACCC sepsis alerts, and education on appropriate scenarios necessitating BCxs. BCxs drawn are reported as BCxs per 1000 days present and skin flora contaminants were defined as BCx with a single occurrence in a 72-hour period of any coagulase-negative *Staphylococci* (non-*lugdunensis*), *Bacillus* (non-*cereus*) spp., *Corynebacterium* (non-*jeikeium*) spp., or *Micrococcus* spp. Descriptive statistics were calculated using mean values and compared using the Wilcoxon rank-sum test.

**Results:**

During the intervention period, diagnostic stewardship efforts decreased BCxs drawn by approximately 56% from a mean of 224 to 99 BCx per 1000 days present (p=0.001) while improving overall BCx positivity rate to 9% compared to 7.3% pre-intervention (p=0.004). Skin flora recovery rate decreased from 2.00 to 1.03 contaminants per 1000 days present (p=0.001), while recovery rate was maintained for Gram-negative organisms at 2.72 per 1000 days present compared to 3.40 (p=0.060). No appreciable changes were seen post-intervention in hospital-wide all-cause mortality or ACCC all-cause mortality for patients with or without sepsis alert.

**Conclusion:**

These data suggest that diagnostic stewardship efforts in a comprehensive cancer center can reduce low-yield blood culturing, resulting in decreased BCx utilization and skin flora contaminant recovery without compromising patient care and safety.

**Disclosures:**

All Authors: No reported disclosures

